# Pemphigoïde gestationis et aplasie cutanée congénitale: à propos d'un cas

**DOI:** 10.11604/pamj.2014.19.47.4914

**Published:** 2014-09-22

**Authors:** Ihssane Hakimi, Youssef Benabdejlil, Abdelhakim Ourraï, Jaouad Kouach, Driss Moussaoui, Mohammed Deyahni

**Affiliations:** 1 Service de Gynécologie-Obstétrique, Hôpital Militaire d'instruction Mohammed V, Rabat, Maroc; 2 Service de Pédiatrie, Hôpital Militaire d'instruction Mohammed V, Rabat, Maroc

**Keywords:** Pemphigoïde gestationis, aplasie cutanée congénitale, cas clinique, Pemphigoid gestationis, aplasia cutis congenita, case report

## Abstract

La pemphigoïde gestationis est une dermatose gravidique apparaissant en général entre la 28^ème^ et la 32^ème^ semaine d'aménorrhée. L’éruption cutanée est prurigineuse, bulleuse ou vésiculopapuleuse et de topographie péri-ombilicale. Outre la clinique, son diagnostic repose sur la biopsie cutanée avec analyse en immunofluorescence directe. Elle se caractérise par sa tendance à récidiver. Le traitement habituel nécessite des dermocorticoïdes. Les conséquences peuvent être maternelles (menace d'accouchement prématuré), fœtales (retard de croissance intra-utérin), et néo-natales (éruption cutanée). Nous rapportons un cas de pemphigoïde gestationis associé à une aplasie cutanée congénitale type 2. S'agit il d'une association fortuite?

## Introduction

La pemphigoïde gestationis est une dermatose bulleuse auto-immune, rare et spécifique de la grossesse. Sa fréquence varie de 1/4000 à 1/50 000 grossesses [1]. Cette pathologie se caractérise par une éruption prurigineuse maculopapuleuse et bulleuse survenant au 2^ème^ ou au 3^ème^ trimestre de la grossesse. Il existe une atteinte de la jonction dermo-épidermique, et seule la biopsie cutanée permet de poser le diagnostic [2]. Alors que l'aplasie cutanée congénitale se définit comme une absence localisée de l’épiderme, du derme et parfois des tissus sous cutanés. Les auteurs rapportent le cas d'une patiente présentant une pemphigoïde gestationis dont le nouveau né présentait une aplasie cutanée congénitale, suggérant une éventuelle relation entre les deux pathologies.

## Patient et observation

**Figure 1:**
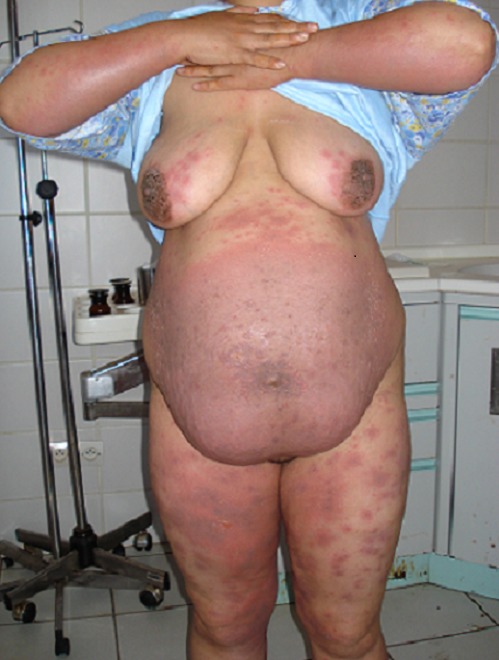
Éruption cutanée vésiculo-papuleuse au niveau de la région péri-ombilicale, bras et cuisses

**Figure 2:**
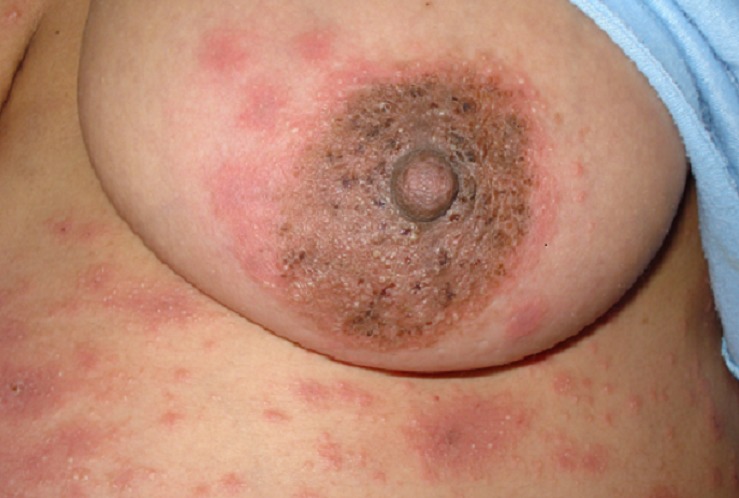
Éruption cutanée vésiculo-papuleuse au niveau de la région péri aréolaire

Mme N.R de 34 ans, multipare sans antécédents pathologiques notables, a mené une grossesse simple à terme au cours de laquelle elle a présenté une éruption cutanée vésiculo-papuleuse et prurigineuse qui n'avait jamais été constaté lors des grossesses précédentes. Ces lésions cutanées prédominaient dans la région péri-ombilicale, les bras, les cuisse et la région péri aréolaire, sans atteinte des muqueuses (Figure 1, Figure 2). Le diagnostic dermatologique d'un pemphigoïde gestationis a été posé après une biopsie cutanée avec examen en immunofluorescence directe qui montrait un infiltrat éosinophile, un œdème sous-épidermique et des dépôts linéaires d'IgG et de fraction C3 du complément le long de la jonction dermo-épidermique. Ce dernier signe est pathognomonique du pemphigoïde gestationis. L'application de corticoïdes locaux avait permis la régression rapide des lésions cutanées.

**Figure 3:**
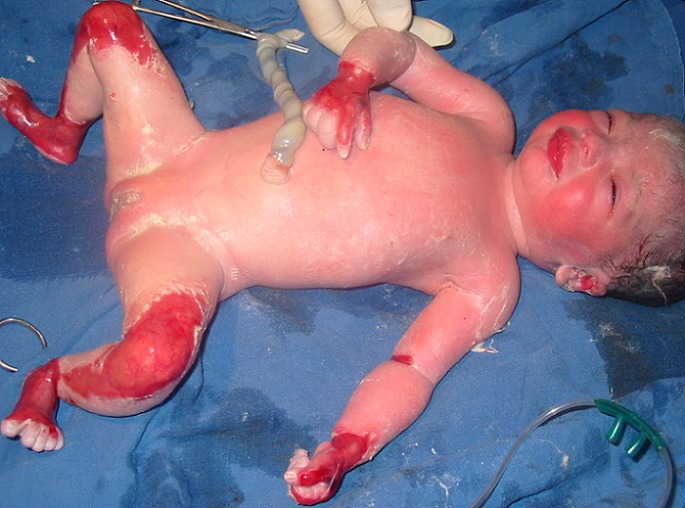
Aplasie cutanée congénitale localisée au niveau des lobes de l'oreille, des coudes, des poignées, des mains, des genoux, des jambes, des chevilles et des pieds

**Figure 4:**
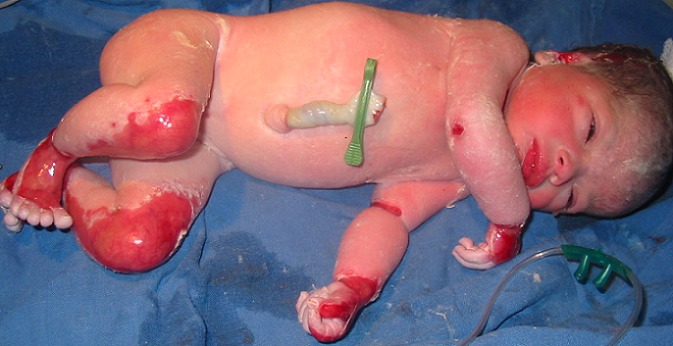
Aplasie cutanée congénitale localisée au niveau des lobes de l'oreille, des coudes, des poignées, des mains, des genoux, des jambes, des chevilles et des pieds

À 28 SA, après l'arrêt du traitement local, la patiente avait de nouveau consulté du fait d'une récidive des lésions cutanées de même topographie ce qui à incité une reprise du traitement. La grossesse était menée à terme avec un suivi régulier ne montrant pas d'anomalies. L'accouchement spontané par voie basse avait eu lieu à 39 SA d'un nouveau né de sexe féminin, poids de naissance de 3000 g qui présentait une aplasie cutanée congénitale localisée au niveau des lobes de l'oreille, des coudes, des poignées, des mains, des genoux, des jambes, des chevilles et des pieds (Figure 3, Figure 4). Le reste de l'examen était sans particularité. Un bilan comprenant les sérologies virales habituelles (cytomégalovirus, rubéole, herpès, varicelle) et bactériennes (notamment listeria et chlamydia), une recherche d'agglutinines irrégulières, un bilan vasculo-rénal (uricémie, numération plaquettaire, bilans hépatique rénal, protéinurie) ainsi qu'un bilan thyroïdien avait été réalisé n'objectivant pas d'anomalies. La régression des lésions cutanées bulleuses chez la patiente a été rapide après une semaine de l'accouchement sans traitement corticoïde.

## Discussion

La pemphigoïde gestationis est une dermatose bulleuse acquise auto-immune survenant en général pendant la grossesse (entre 28 et 32 SA) mais aussi dans le post-partum immédiat ou en cas de môle hydatiforme voire de choriocarcinome. Son association à d'autres pathologies auto-immunes, notamment thyroïdiennes, est possible. Un prurit précède l’éruption d'une à quatre semaines. Classiquement, la topographie de cette éruption est péri-ombilicale avec une extension centrifuge, mais la face est souvent respectée. Une atteinte des muqueuses est possible. L’évolution de la pemphigoïde comporte une amélioration clinique dans les six à huit semaines précédant l'accouchement suivie d'une exacerbation transitoire dans le postpartum immédiat dans 75 à 85% des cas [2]. Parfois une pigmentation maculaire cicatricielle persiste.

Une des caractéristiques de la pemphigoïde est sa tendance à récidiver à chaque grossesse. La survenue de cette pathologie est alors habituellement plus précoce et plus sévère. Certaines poussées ont été décrites lors de la prise d'oestroprogestatifs. Ces faits méritent d’être précisés à une patiente atteinte d'un premier épisode de pemphigoïde gestationis. La physiopathologie, assez complexe, comporte une dysrégulation immunitaire entre l'unité fœtoplacentaire et la mère survenant sur un terrain génétique prédisposé (haplotype HLA DR3-DR4). Il se produit une baisse de la tolérance maternelle vis-à-vis du fœtus créant alors une réaction contre les antigènes placentaires, en partie d'origine paternelle. Ces antigènes sont des polymères ayant des particules communes aux antigènes de la jonction dermo-épidermique. Les anticorps synthétisés détruisent la membrane basale, ce qui conduit à la formation de bulles sous-épidermiques. L’étude en immunofluorescence directe est pathognomonique: elle permet de mettre en évidence des dépôts linéaires d'IgG et de fraction C3 du complément le long de la jonction dermo-épidermique. En immunofluorescence indirecte on recherche un auto-anticorps sérique circulant (Herpès gestationis factor) de type IgG. Sa spécificité est de 100% mais la sensibilité n'est que de 75%. Le diagnostic différentiel d'une pemphigoïde gestationis est initialement celui d'un prurit isolé puis celui de tout prurit associé à des lésions dermatologiques pendant la grossesse.

Il convient avant tout de réaliser un examen clinique complet. Celui ci permettra d'envisager les différentes étiologies devant un prurit nu ou associé à une dermatose. En cas de prurit nu, le premier diagnostic à évoquer est la cholestase gravidique qui reste un diagnostic d’élimination. la biopsie cutanée et l'analyse immunofluorescence directe permettront de différencier pemphigoïde gestationis des autres dermatoses gravidiques (dermatite herpétiforme, rash toxémique, etc.). Dans les rares cas de lupus bulleux, l'immunofluorescence directe vêt un aspect différent: les dépôts d'immunoglobulines sont discontinus sur la membrane basale. Le traitement habituel de la pemphigoïde gestationis est la corticothérapie par voie locale. Dans les formes sévères, un recours aux corticoïdes par voie générale est nécessaire. D'autres alternatives thérapeutiques sont possibles (immunoglobulines intraveineuses, plasmaphérèses ou sulfamides) mais leur utilisation reste exceptionnelle [3]. Les conséquences maternelles de la pemphigoïde gestationis sont dominées par le risque de récidive lors des grossesses ultérieures (plus précoces et plus sévères) [2] mais également par les menaces d'accouchement prématuré (taux variable selon les auteurs de 0 à 23%) [1]. La contraception par oestroprogestatifs expose à un risque de récidive [4].

Les conséquences fœtales sont multiples: retard de croissance intra-utérin [5] ou mort fœtale in utero (0 à 7,7% des cas) [1, 6]. Après la naissance, des lésions néonatales précoces survenant avant le 7 e jour de vie peuvent exister. Elles sont constatées dans 5 à 10% des cas; leur fréquence dépend du passage transplacentaire des auto-anticorps qui est faible. Ces atteintes néonatales sont essentiellement cutanées (lésions bulleuses identiques à celles de la mère [1, 7]) mais aussi neurologique dans de rares cas (hémorragie cérébrale [8, 9]). Dans notre cas il s'agit plutôt d'une aplasie cutanée congénitale. L'aplasie cutanée congénitale est de diagnostic généralement néonatal par la découverte d'une perte de substance cutanée isolée. La prédominance céphalique des lésions (73%). Alors que l'association des malformations associées est de 37,1%, l'atteinte osseuse sous-jacente est de 9% et les syndromes malformatifs associées est de 13,7%. Le problème essentiel posé au clinicien est de savoir si l'aplasie cutanée congénitale est réellement isolée ou s'il s'agit de l'un des signes clinique d'un syndrome malformatif [10, 11]. L'analyse de la littérature ne retrouve aucun cas de pemphigoïde gestationis associé à l'aplasie cutanée congénitale. Dans le cas de notre observation, est ce qu'il s'agissait d'une association fortuite.

## Conclusion

La pemphigoïde gestationis est rare et son association à l'aplasie cutanée congénitale semble fortuite. Une relation entre les deux pathologies semble difficiles à établir en raison de l'exceptionnelle survenue de telles associations.
